# Peripheral mast cells derive the effects of acupuncture in Parkinson’s disease

**DOI:** 10.3389/fnagi.2024.1376756

**Published:** 2024-06-24

**Authors:** Ju-Young Oh, Sun-Jeong Bae, Jeong-Yeon Ji, Tae-Yeon Hwang, Suhwan Ji, Ji-Yeun Park, Seung-Nam Kim, Yeonhee Ryu, Min-Ho Nam, Hi-Joon Park

**Affiliations:** ^1^Department of Anatomy and Information Science, College of Korean Medicine, Kyung Hee University, Seoul, Republic of Korea; ^2^Laboratory of Acupuncture and Neuro Medicine, Acupuncture and Meridian Science Research Center (AMSRC), Kyung Hee University, Seoul, Republic of Korea; ^3^Department of Epidemiology, Harvard T.H. Chan School of Public Health, Boston, MA, United States; ^4^Department of Clinical Korean Medicine, College of Korean Medicine, Kyung Hee University, Seoul, Republic of Korea; ^5^College of Korean Medicine, Daejeon University, Daejeon, Republic of Korea; ^6^College of Korean Medicine, Dongguk University, Goyang, Republic of Korea; ^7^Clinical Medicine Division, Korea Institute of Oriental Medicine, Daejeon, Republic of Korea; ^8^Brain Science Institute, Korea Institute of Science and Technology, Seoul, Republic of Korea; ^9^Department of KHU-KIST Convergence Science and Technology, Kyung Hee University, Seoul, Republic of Korea

**Keywords:** acupuncture, Parkinson’s disease, peripheral mechanism, mast cell, free nerve endings

## Abstract

This research investigates the peripheral mechanisms of acupuncture in treating Parkinson’s disease (PD), a progressive neurodegenerative disorder marked by motor impairments. While the central mechanisms of acupuncture have been extensively studied, our focus lies in the peripheral mechanisms at the acupoints, the sites of acupuncture signal initiation. Employing a PD model, we analyzed the local responses to acupuncture stimulation at these points. Our key finding was a significant elevation in both the number and activity of mast cells (MCs) in the peripheral tissues following acupuncture. Intriguingly, pre-treatment with an MC stabilizer diminished the acupuncture’s therapeutic effects on PD symptoms. Similarly, local anesthesia with lidocaine at the acupoints attenuated the symptom improvement typically observed with acupuncture. Meanwhile, the augmentation of MC activity induced by acupuncture was significantly impeded by cromolyn, an MC stabilizer, but remained unaffected by lidocaine. This finding suggests that MC activity is a more upstream regulator of acupuncture effects compared to nerve conduction. This study provides groundbreaking insights into the initiation and transmission of acupuncture signals, highlighting the significant role of peripheral MC modulation in PD treatment.

## 1 Introduction

Parkinson’s disease (PD) is the second most common neurodegenerative disorder characterized by the progressive loss of dopaminergic neurons in the substantia nigra (SN) (Bloem et al., 2021). Clinical diagnosis is primarily based on the manifestation of motor deficits, including stiffness, resting tremor, bradykinesia, and postural instability (Albin et al., 1989). In addition to this, patients also experience a range of non-motor symptoms such as mood disorders, cognitive impairment, autonomic dysfunctions, sleep disorders, and gastrointestinal disorders (Chaudhuri and Schapira, 2009; Schapira et al., 2017). Current treatments for PD are usually used to replenish neuronal dopamine to improve movement disorders (Lane, 2019; Armstrong and Okun, 2020). Unfortunately, current strategies only improve the patients’ symptoms for a limited period of time and also show resistance and development of induced dyskinesias during treatment (Oertel, 2017). Given the wide range of symptoms and side effects of current treatments, neuroprotective and/or disease-modifying therapies in the treatment and management of PD remain limited.

Acupuncture is an important complementary therapy rooted in traditional East Asian medicine. It is widely applied and is a safe, convenient, and cost-effective approach with minimal side effects (Yu et al., 2019). By stimulating specific acupoints on the skin and muscles, acupuncture induces morphological deformation in the surrounding acupoints, which is believed to initiate acupuncture signals. It is speculated that these changes play crucial role in initiating acupuncture signals by inducing various cellular and molecular responses, including local responses such as ERK activation in keratinocytes and fibroblasts, peripheral adenosine release, and TRPV1 in the subepidermal nerve fibers (Abraham et al., 2011; Park et al., 2021). Adenosine A1 receptors in nerve endings and PROKR2 nerve fibers also contribute significantly to mediating the effects of acupuncture (Goldman et al., 2010; Liu et al., 2021).

Especially, recent acupuncture studies have reported that acupuncture stimulation can activate and induce degranulation peripheral mast cells (MCs) (Ding et al., 2018; Li et al., 2022). Research suggests that MC degranulation triggered by acupuncture plays a significant role in pain relief, as MCs are involved in immediate hypersensitivity reactions and pain modulation (Bae et al., 2021). Interestingly, acupuncture-induced MC degranulation in the skin contributes to pain relief, and pretreatment with cromolyn, an MC stabilizer, diminishes this analgesic effect (Huang et al., 2018). The signals initiated by acupuncture are transmitted to the central nervous system through activated afferent nerve fibers, leading to the regulation of various physiological functions. Neurotransmitters such as opioid peptides, serotonin, norepinephrine, and dopamine are released from the spinal cord and brain, producing complex actions that suppress pain. Although the peripheral role of MCs in acupuncture has been mainly studied in the context of pain, recent research suggests their involvement in broader acupuncture mechanisms. These findings shed light on the significance of peripheral MCs in acupuncture stimulation beyond pain studies.

To date, acupuncture has been widely used in the treatment of various neurodegenerative diseases, including PD. The efficacy of acupuncture in PD has been investigated in both patients with PD and animal models. Numerous studies suggested that acupuncture could enhance striatal dopamine levels and address neurotransmitter imbalances (Jeon et al., 2008; Kim et al., 2014; Oh et al., 2022). Furthermore, several animal studies have shown that acupuncture protects against neuronal death by inhibiting neuroinflammatory responses, reducing apoptosis, and activating neurotrophic factors (Park et al., 2015; Jang et al., 2020). However, research on the mechanism of acupuncture treatment for PD has predominantly focused on central mechanisms, while the exploration of peripheral mechanisms has been relatively understudied and limited in scope. Therefore, it remains unclear whether the therapeutic effects of acupuncture treatment in PD are associated with peripheral MCs and whether a relationship exists between MCs and nerves.

Here, we aimed to confirm the involvement of MCs in the peripheral mechanism underlying the amelioration of both motor and non-motor symptoms in a mouse model of PD through acupuncture-triggered local acupoint responses. In addition, we investigate to establish a causal relationship between peripheral nerve or MC activity and the conversion of acupuncture stimulation into effective acupuncture signals.

## 2 Materials and methods

### 2.1 Animals

All procedures were conducted in accordance with the National Institutes of Health guidelines and approved by the Institutional Animal Ethical Committee, Kyung Hee University (Seoul, Korea, Approval Number KHSASP-23-398). Male C57BL/6 J mice were used from 8 weeks old. Mice were co-housed at 22 ± 2°C, with a relative humidity of 55 ± 15%, and provided with food and water *ad libitum*. All mice were housed in a temperature-controlled facility with a 12-h light/dark cycle (lights on at 8:00 a.m., lights off at 8:00 p.m.). All mice were randomly allocated to the different experimental groups.

### 2.2 Experimental timeline and grouping

This study consists of two experiments. The first aims to investigate the involvement of MCs or nerves in the effects of acupuncture treatment in the MPTP model. In this experiment, a total of 5 groups were assigned, each consisting of 6 mice: a naïve group (Naïve), a group injected with MPTP for 5 days (MPTP), an MPTP injection group receiving acupuncture treatment (MPTP + Acu), an MPTP injection group receiving pretreatment with the MC stabilizer cromolyn before acupuncture treatment (MPTP + Cromolyn + Acu), and an MPTP injection group receiving pretreatment with the local anesthetic lidocaine before acupuncture treatment (MPTP + Lidocaine + Acu). The second aims to confirm the precedence between MCs of nerves. In this experiment, mice were randomly assigned to each group, with 6 mice per group. The groups included: a naïve group that received no treatment (Naïve), a group that received acupuncture stimulation for 15 min (Acu), a group that was pretreated with cromolyn before acupuncture stimulation (Cromolyn + Acu), and a group that was pretreated with lidocaine before acupuncture stimulation (Lidocaine + Acu). All mice were sacrificed at the same time point after 15 min of acupuncture stimulation.

### 2.3 Acupuncture treatment

Acupuncture was performed at the GB34 acupoint by inserting as stainless-steel acupuncture needle (15 mm in length and 0.20 mm diameter; Haeng-lim-seo-weon Acuneedle Co., Seoul, Korea) about 3 mm depth. The GB34 acupoint is located at the intersection point of lines from the anterior borders to the head of the fibula. These inserted needles were then turned at a rate of two spins per second for 30 s and removed immediately. Treatment was performed accurately and quickly to minimize stress on the mice. Also, mice in all groups were mildly immobilized by holding their necks, with the head in an upright position for 30 s to give the same immobilization stress as the acupuncture group.

### 2.4 Drug administration

All 1-methyl-4-phenyl-1,2,3,6-tetrahydropyridine (MPTP) experiments used the subchronic regimen consisting of a daily intraperitoneal (i.p.) injection of MPTP (30 mg/kg per day, Sigma-Aldrich, Missouri, United States; 23007-85-4) for five consecutive days. Additionally, 5 min before acupuncture stimulation, Cromolyn sodium salt (0.02 g ml^−1^, 10 μL, Sigma-Aldrich; 15826-37-6) to stabilize MC degranulation or 10 μL lidocaine (lidocaine-HCl 400 mg; Huons, Korea) to block the axonal reflex with a local anesthetic were intradermally injected at GB34.

### 2.5 Behavior tests

#### 2.5.1 Cylinder test

To assess the spontaneous explorative behavior in a new environment, a cylinder test was performed. Prior to the experiment, mice were placed in a transparent plastic cylinder (12 cm in diameter × 20 cm tall) for 1 min. After adaptation, when mice try to explore different areas of the cylinder by standing on their hindlimbs and leaning with the forelimbs on the cylinder wall, the cylinder wall touches (numbers) were counted by observers for 3 min.

#### 2.5.2 Rotarod test

An accelerated rotarod test was conducted to evaluate the coordination and balance of motor function. On the day of testing, mice were kept in their home cages and acclimated to the testing room for at least 30 min. A 2 min acclimation session at 2 rpm was performed on the first day before the test phase. Rotarod testing involves placing mouse on a rotating bar and determining the length of time that they can retain their balance while the rotation speed increases over 480 s. The speed is increased from 3.5 rpm to 35 rpm, reaching maximum speed at 5 min. Each trial was terminated when the mouse fell off. The latency to fall from the rotating rod was scored by automatic timers and falling sensors on the rotarod.

#### 2.5.3 Pole test

The pole test was to measure bradykinesia in the PD model. The instrument consists of a 55 cm-high pole and 1.3 cm in diameter. The mouse was placed head upward near the top of the pole, and the time taken for the mouse to reach the floor was determined.

#### 2.5.4 Elevated plus maze test

The EPM test was used to determine anxiety-related behavior in mice. As animals explored the maze for 5 min, their behavior was recorded using a video camera located on the ceiling above the center of the maze and this video was relayed to the S-MART program (PanLab, Barcelona, Spain). Arm entry was defined as entering an arm with all four paws. This apparatus consisted of two open arms and two closed arms was connected central platform. At the beginning of each trial, animals were placed at the center of the maze, facing a closed arm. Anxiety reduction, as manifested by open arm exploration in EPM test, was defined as an increase in the number of open arms compared to total entries into either open arm or closed arm, and an increase in the percentage of time spent in open arms. Total hours spent on the arms. Total arm entries were also used as an indicator of changes in the locomotor activity of the rats. The anxiety index was calculated as follows: Anxiety index = 1 − [(Time spent in the open arms / Total time on the maze) + (Number of entries to the open arms / Total exploration on the maze)] / 2.

#### 2.5.5 Open field test

The OF test was used to measure the spontaneous locomotor activity and anxiety-like behavior of the animals. All mice were habituated to the testing room for 1 h immediately before the test. Each of them was individually housed in a rectangular container in a dark room (60 × 60 × 30 cm). This material was used to provide the best contrast to the black mice in a dimly lit room. The room was equipped with a video camera above the center of the room so that the locomotor activities of the mice could be measured. Locomotor activities were assessed using the distance that the mice traveled and monitored by a computerized video-tracking system using the S-MART program (Pan Lab Co., Barcelona, Spain). The distance traveled in the container was recorded for 5 min.

### 2.6 Immunostaining

Mice were deeply anesthetized and transcardially perfused with phosphate-buffered saline (PBS) followed by 4% paraformaldehyde (PFA) in 0.2 M phosphate buffer. The brains were removed, postfixed in 4% PFA, and cryoprotected in 10, 20, and 30% sucrose at 4°C for 3 days. Brains were divided into 30 μm thick sections on a cryostat microtome. For studies of tyrosine hydroxylase (TH)-positive fiber, tissues were incubated in rabbit polyclonal anti-TH primary antibody (1:1,000, Santa Cruz Biotechnology; sc-14007) for 24 h at 4°C. After washing three times in PBS, the sections were incubated in goat anti-rabbit Alexa Fluor 488 (1:1,000; Thermo Fisher Scientific) secondary antibody for 1 h at room temperature. Then, they were washed in PBS.

### 2.7 Imaging and quantification

Fluorescent images of TH-positive neurons in the SN were obtained using a confocal scanning microscope (Olympus FV-1000 laser) with Image J version 1.50i software (National Institutes of Health, Bethesda, MD, United States). To quantify changes in the number of TH-positive neurons in the SN regions, the number of TH-labeled cells was determined in every sixth section in a series of 30 μm coronal sections. The total positive cell number was multiplied by the virtual counted number with the reference factors (1/6 sections analyzed; 1/16 counting area; and the tissue shrink factor). TH-positive fibers in the striatum were imaged and quantified by measuring the optical density of TH immunofluorescence using Image J software (NIH, MD, United States), and the value was reported as the optical density.

### 2.8 Western blotting

Brain samples were homogenized in 200 μL of lysis buffer (CyQUANT; Invitrogen, Eugene, OR, USA) including phosphatase inhibitor cocktail tablets and protease inhibitor cocktail tablets. After homogenization, the samples were centrifuged at 12,000 rpm for 15 min at 4°C and the supernatants were collected. The amount of protein was measured using the BCA assay. For Western blot analysis, equal protein concentrations (10 μg of total protein) were separated by a 10% sodium dodecyl sulfate-polyacrylamide gel electrophoresis (SDS-PAGE) and then transferred to a PVDF membrane (Millipore, Billerica, MA, United States). The membrane was blocked in 5% skim milk in Tris buffered saline containing 0.1% Tween-20 (TBS-T) and incubated with the primary antibodies overnight at 4°C. The primary antibodies: TH (Santa Cruz Biotechnology; sc-14007), GFAP (Invitrogen; #14-9892-82), Iba-1 (Millipore; #MABN92), IL-6 (Santa Cruz Biotechnology; sc-57315), IL-1β (Cell signaling; #12242), TNF-α (Santa Cruz Biotechnology; sc-52746), HDC (abcam; ab37291), Chymase (abcam; ab2377), Tryptase (abcam; ab2378), and β-actin (Sigma-Aldrich; #A1978). Then, the membrane was incubated with the secondary horseradish peroxidase-conjugated goat anti-rabbit antibody (Pierce, Rockford, IL, United States) or mouse (Thermo Scientific; PA1-30355) antibodies. The membrane was visualized using a chemiluminescence kit (Super Signal West Pico; Pierce, Rockford, IL, United States). The signal intensities from the immunoblots were analyzed by densitometry.

### 2.9 Histochemical staining

The needles were removed after the paraffinization of the skin samples and before paraffin embedding. Tissue blocks were cut parallel to the needle axis at 8 μm thickness using a manual rotary microtome (Shandon Finesse 325; Thermo Fisher Scientific Inc.). Every fifth slide with a needle track label was stained with Masson trichrome-counterstained (Masson Trichrome Stain Kit, Nova Ultra^™^; IHC World, Ellicott City, MD, United States), which stains collagen as blue. To investigate the MC degranulation, toluidine blue staining was performed on formalin-fixed, paraffin-embedded 8 μm skin sections. Briefly, sections were deparaffinized, rehydrated, and stained with toluidine blue working solution (pH 2.0–2.5).

### 2.10 Statistical analysis

All data were graphed and analyzed using GraphPad Prism 9 software (GraphPad Software Inc., San Diego, CA, United States). To compare multiple groups, the statistical analysis was performed using a one-way analysis of variance (ANOVA) with Tukey’s *post hoc* test. All the data were expressed as the mean ± standard error of the mean. The significance level is represented as asterisks (^∗^*p* < 0.05, ^∗∗^*p* < 0.01, ^∗∗∗^*p* < 0.001).

## 3 Results

### 3.1 Peripheral MCs contribute to the effects of acupuncture in the MPTP model

**Figure 1:**
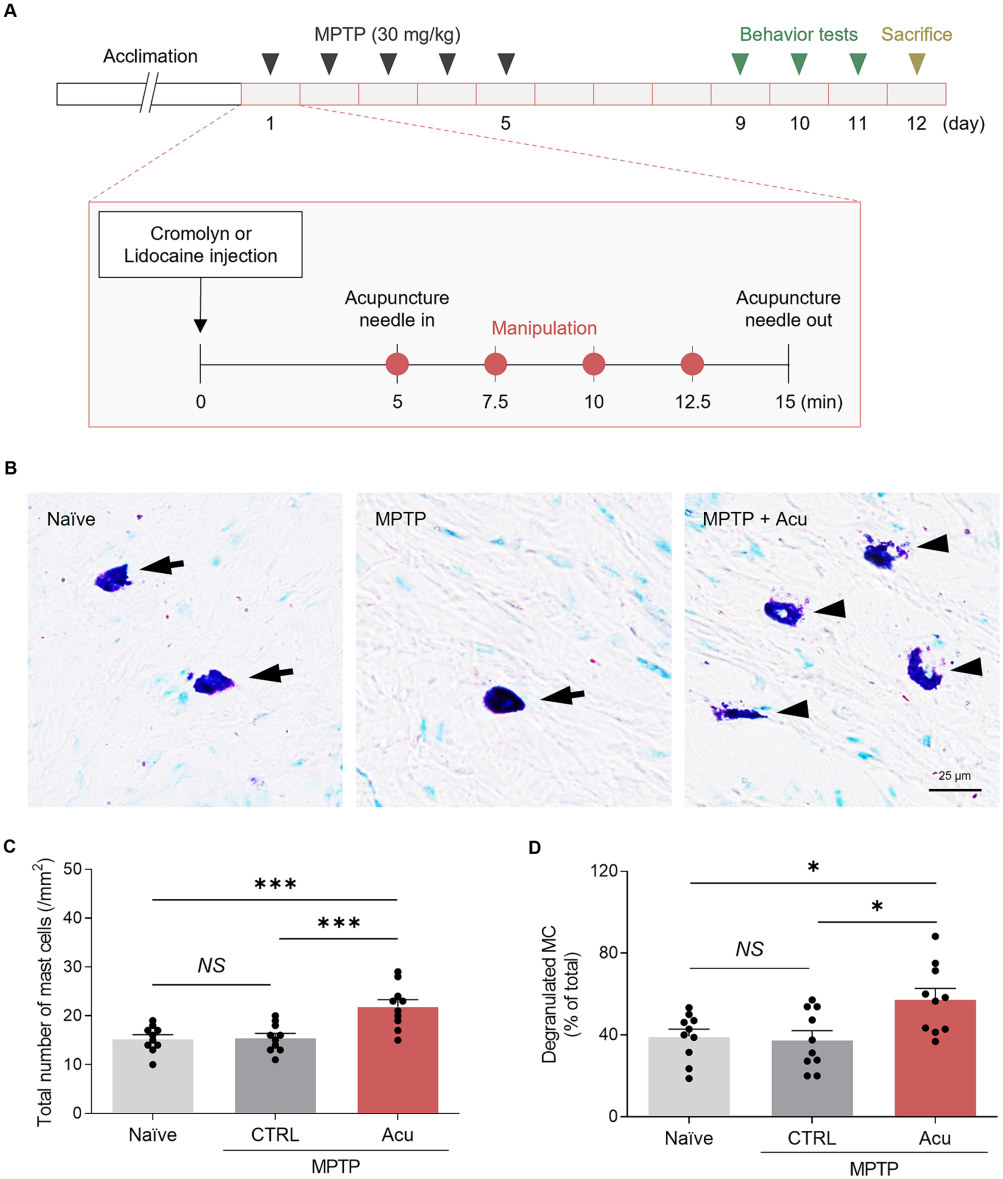
Increased number and degranulation of peripheral MCs associated with therapeutic effects of acupuncture in PD mice. **(A)** Experimental timeline for therapeutic effects in a MPTP model. MPTP was administered intraperitoneally for 5 days, while inhibitor or acupuncture treatments were conducted for 12 days. Behavioral experiments were performed starting from day 9, and sacrifice was conducted on day 12. **(B)** Representative images of toluidine blue staining for MCs. **(C,D)** Quantification of number and degranulated peripheral MCs (*N* = 5 mice and *n* = 10 cells per group). ^***^p < 0.001, ^*^p < 0.05, NS, non-significant. All data are presented as mean ± SEM.

We first ascertained whether peripheral MCs were involved in acupuncture stimulation at GB34 in an MPTP-induced PD mice model (Figure 1A). The number of MCs in the acupoint was counted using toluidine blue staining to label MCs, and the degranulation ratio was calculated as the degranulated MCs versus the total number of MCs (Figures 1B–D). Intact MCs were elliptical or fusiform in shape with clear edges (cells indicated by arrowhead). Degranulated MCs were elliptical or fusiform with incomplete and blurred edges or irregularly broken sheets (cells indicated by arrows). We found that in the GB34 acupoint, MC numbers and degranulation were also increased by acupuncture stimulation (one-way ANOVA with *post hoc* Tukey’s tests: total number of MCs, *F*_2, 27_ = 11.87, *p* < 0.001; Degranulated MC, *F*_2, 27_ = 5.853, *p* = 0.008; Figures 1C,D; Supplementary Figure S1). Thus, these results showed the involvement of MCs in acupuncture stimulation in an MPTP mice model.

### 3.2 Peripheral MCs and nerves play a role in the motor improvement effects of acupuncture in the MPTP model

**Figure 2:**
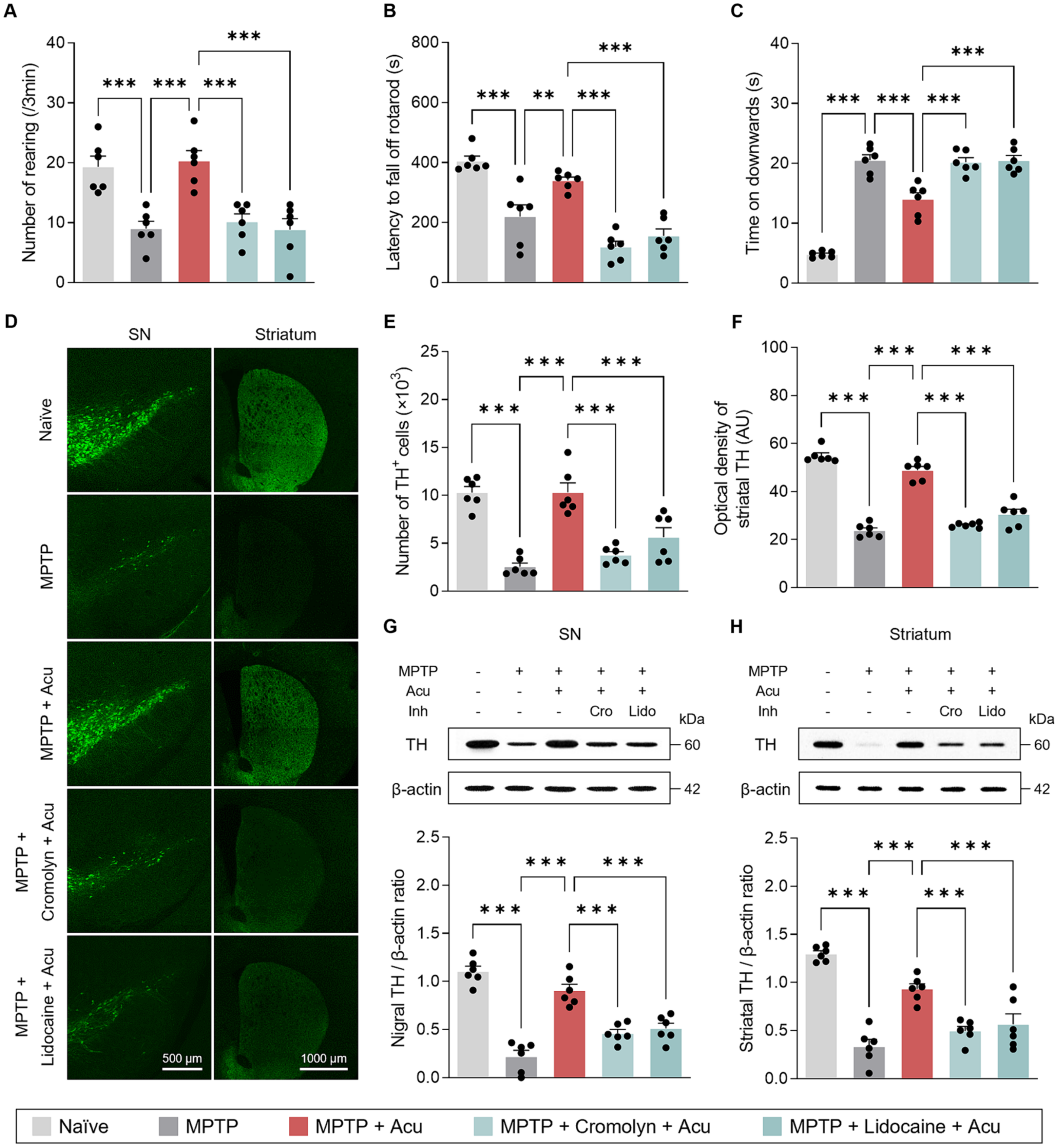
Peripheral MCs and nerves associated with motor symptoms improvement and neuroprotective effects of acupuncture in PD mice. **(A–C)** Motor symptoms improvement effects of acupuncture assessed by cylinder, rotarod, and pole tests. **(D)** Representative images of TH-stained SN and striatum tissues. **(E)** Quantification of TH-positive cells in SN. **(F)** Quantification of TH optical density in striatum. **(G,H)** Representative western blot image and quantification of TH expression levels in the SN and striatum. ^***^p < 0.001. *n* = 6 per group. All data are presented as mean ± SEM.

It is well known that acupuncture stimulation at GB34 acupoint induces ameliorative effects on movement disorders in PD animal models and patients (Jeon et al., 2008; Oh et al., 2022). Consistent with previous observations, motor deficits in the subchronic MPTP model were ameliorated by acupuncture at GB34 in the behavioral tests (one-way ANOVA with *post hoc* Tukey’s tests: cylinder, *F*_4, 25_ = 13.1, *p* < 0.001; rotarod, *F*_4, 25_ = 26.9, *p* < 0.001; pole test, *F*_4, 25_ = 68.0, *p* < 0.001; Figures 2A–C). Interestingly, these improvement effects of acupuncture on motor impairment were blocked by pretreatment with cromolyn, an MC stabilizer, or lidocaine, a local anesthetic, respectively. In other words, the MC changes in acupoints by acupuncture stimulation and peripheral nerve endings are involved in acupuncture signals of therapeutic effects that improve parkinsonian motor deficits.

As the MCs and peripheral nerves were involved in the motor improvement effects caused by acupuncture stimulation, we further investigated the neuroprotective effects in the SN and striatum. From the fluorescence staining, the significant and progressive loss of TH-positive dopaminergic neurons in the SN and striatum caused by MPTP treatment was recovered by acupuncture, but the treatment effect was blocked by pretreatment with cromolyn or lidocaine (one-way ANOVA with *post hoc* Tukey’s tests: SN, *F*_4, 25_ = 24.5, *p* < 0.001; striatum, *F*_4, 25_ = 100, *p* < 0.001; Figures 2D–F). Also, from the western blot, the expression levels of TH recovered by acupuncture were blocked by pretreatment with cromolyn or lidocaine (one-way ANOVA with *post hoc* Tukey’s tests: SN, *F*_4, 25_ = 41.0, *p* < 0.001; striatum, *F*_4, 25_ = 33.1, *p* < 0.001; Figures 2G,H). These results indicate that MC changes in acupoints and peripheral nerves are involved in the improvement of dopaminergic neurons by acupuncture treatment in the MPTP model.

### 3.3 Peripheral MCs and nerves play a role in the non-motor improvement effects of acupuncture in the MPTP model

**Figure 3:**
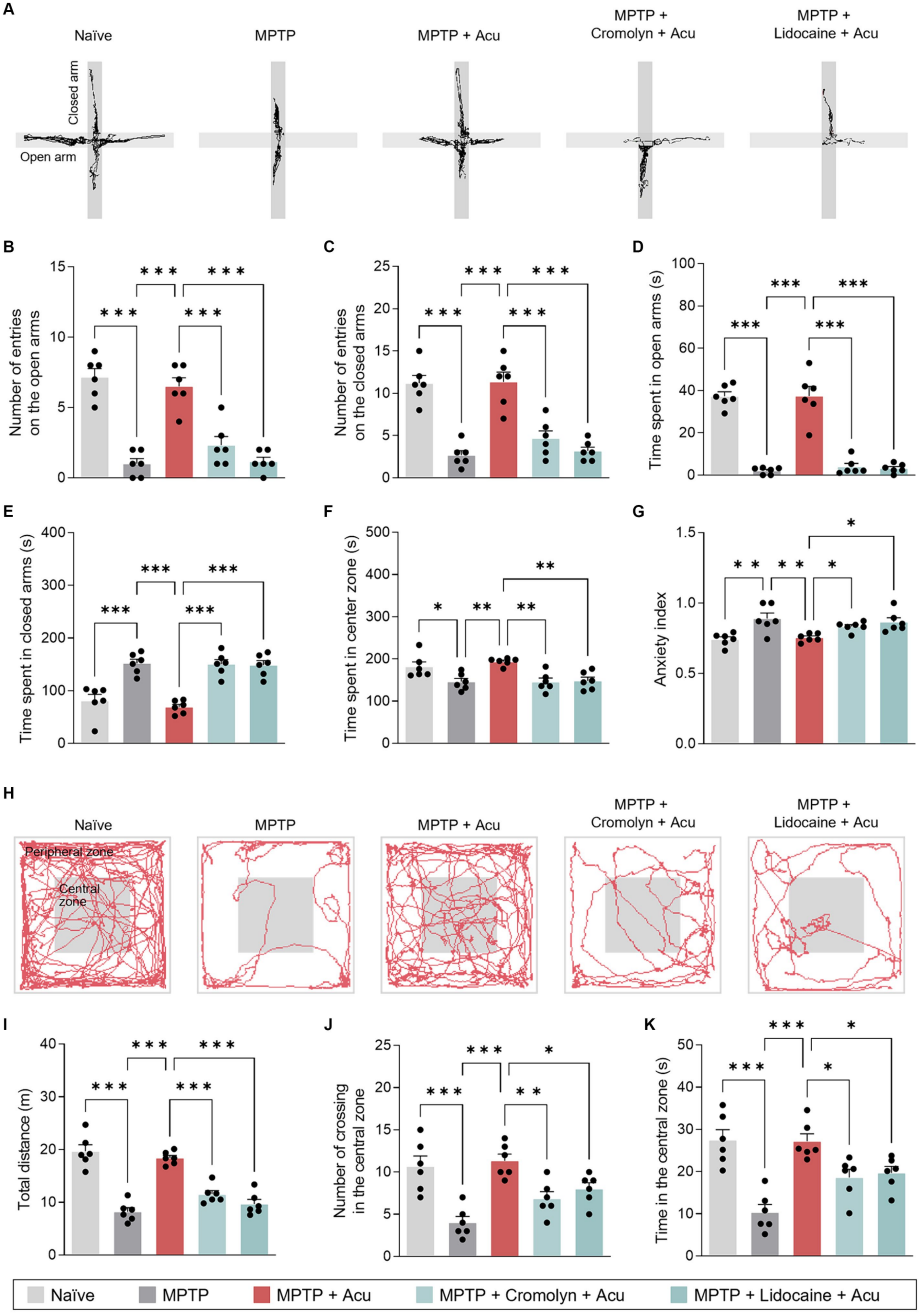
Peripheral MCs and nerves associated with non-motor symptoms improvement effects of acupuncture in PD mice. **(A)** Track plots of a single mouse from each group of EPM test. **(B–G)** Number of entries or time spent on the open and closed arms and anxiety index assessed by EPM test. **(H)** Representative track plots a single mouse from each group of the OF test. **(I–K)** Total distance, number of crossing in the central zone, and time in the central zone assessed by OF test. ^***^p < 0.001, ^**^p < 0.01, ^*^p < 0.05. *n* = 6 per group. All data are presented as mean ± SEM.

In addition to the well-known motor deficits in PD, key features of PD also include non-motor symptoms involving anxiety-like behavior. We next assessed anxiety using the OF and EPM tests to determine whether the involvement of peripheral MCs and nerves by acupuncture stimulation at GB34 was also associated with non-motor symptoms. On the EPM, the number of entries in the open and closed arms significantly increased with acupuncture stimulation compared to the MPTP-induced mice (one-way ANOVA with *post hoc* Tukey’s tests: the number of entries on the open arms, *F*_4, 25_ = 32.8, *p* < 0.001; the number of entries on the closed arms, *F*_4, 25_ = 25.9, *p* < 0.001; time spent in open arms, *F*_4, 25_ = 59.5, *p* < 0.001; time spent in closed arms, *F*_4, 25_ = 21.8, *p* < 0.001; time spent in center zone, *F*_4, 25_ = 7.23, *p* < 0.001; Figures 3A–F). These effects were inhibited by pretreatment with cromolyn or lidocaine. Moreover, the acupuncture group exhibited a statistically significant lower anxiety index compared to the MPTP group, while the group pretreated with cromolyn or lidocaine showed a statistically significant increase in anxiety compared to the acupuncture group (one-way ANOVA with *post hoc* Tukey’s tests: *F*_4, 25_ = 6.78, *p* < 0.001; Figure 3G).

The OF test was performed to evaluate locomotor and anxiety-like behaviors. The acupuncture stimulation group showed a significant increase in total distance traveled and the number and percentage of time spent in the central zone compared to MPTP-induced mice (one-way ANOVA with *post hoc* Tukey’s tests: total distance, *F*_4, 25_ = 37.5, *p* < 0.001; number of crossings in the central zone, *F*_4, 25_ = 11.3, *p* < 0.001; time in the central zone, *F*_4, 25_ = 13.3, *p* < 0.001; Figures 3H–K). As indicated by the results, acupuncture is effective in improving both motor and non-motor symptoms, suggesting the involvement of peripheral MCs and nerves at acupoints in producing these effects.

### 3.4 Peripheral MCs and nerves play a role in anti-neuroinflammatory effects of acupuncture in the MPTP model

**Figure 4:**
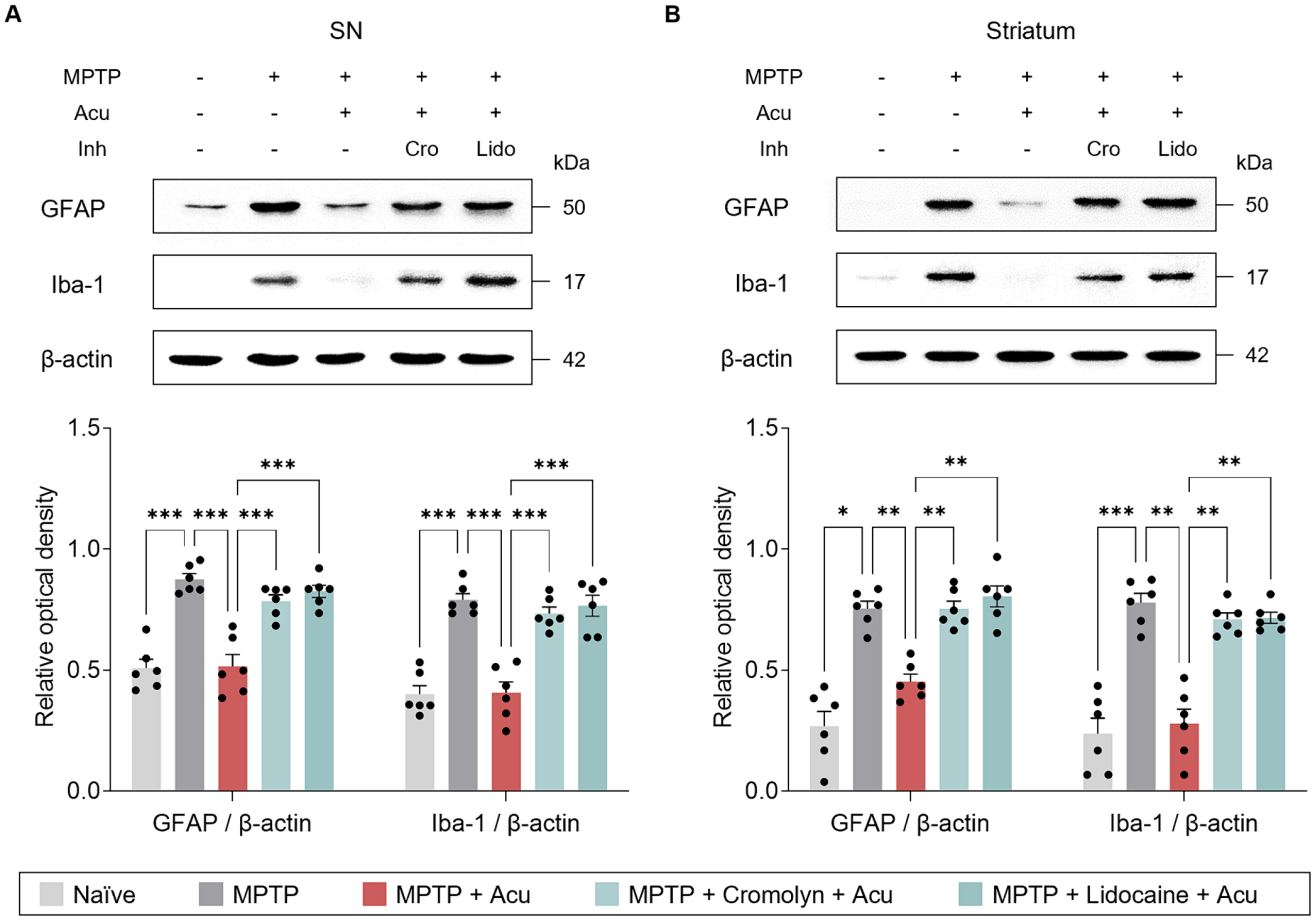
Peripheral MCs and nerves associated with inhibitory effects of acupuncture on glial cells in PD mice. **(A)** Representative bands of GFAP and Iba-1, as well as their quantification by western blotting analysis in the SN. **(B)** Representative bands of GFAP and Iba-1, as well as their quantification by western blotting analysis in the striatum. ^***^p < 0.001, ^**^p < 0.01, ^*^p < 0.05. *n* = 6 per group. All data are presented as mean ± SEM.

Inflammation influences the pathogenesis and progression of PD, and acupuncture treatment at GB34 has been reported to have anti-inflammatory effects. Therefore, to investigate whether MCs are involved in these effects, Iba-1 and GFAP were measured in the striatum and SN to determine the expression levels of microglia and astrocytes, which are important immune cells in the brain (Figure 4). As a result, as in the previous study, acupuncture stimulation on GB34 has anti-inflammatory effects, but these effects inhibited with cromolyn or lidocaine pretreatment in the SN (one-way ANOVA with *post hoc* Tukey’s tests: GFAP, *F*_4, 25_ = 27.8, *p* < 0.001; Iba-1, *F*_4, 25_ = 29.4, *p* < 0.001; Figure 4A) and striatum (one-way ANOVA with *post hoc* Tukey’s tests: GFAP, *F*_4, 25_ = 32.6, *p* < 0.001; Iba-1, *F*_4, 25_ = 33.4, *p* < 0.001; Figure 4B).

**Figure 5:**
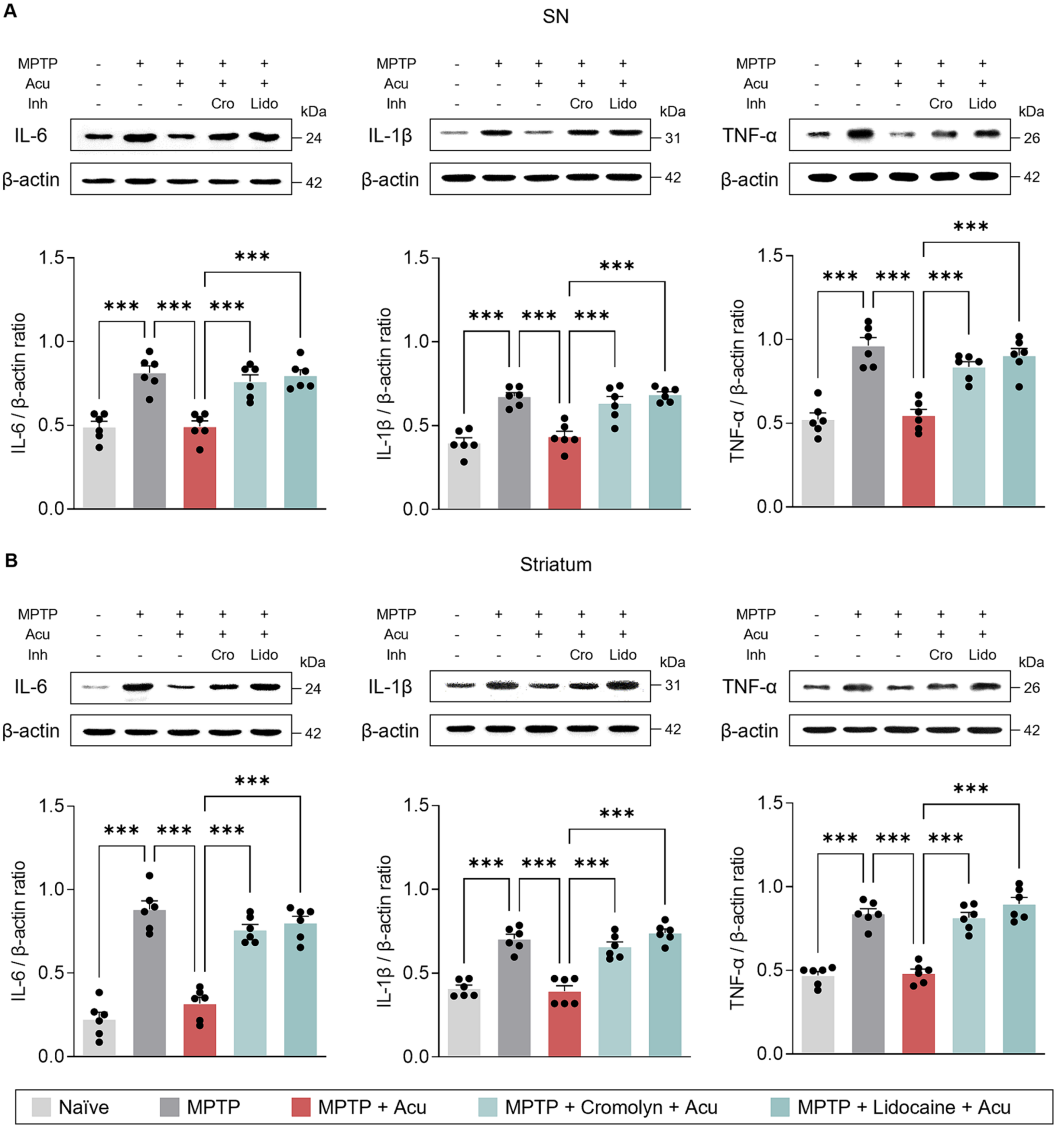
Peripheral MCs and nerves associated with inhibitory effects of acupuncture on inflammatory cytokines in PD mice. **(A)** Representative bands of IL-6, IL-1β, and TNF-α, as well as their quantification by western blotting analysis in the SN. **(B)** Representative bands of IL-6, IL-1β, and TNF- α, as well as their quantification by western blotting analysis in the striatum. ^***^p < 0.001. *n* = 6 per group. All data are presented as mean ± SEM.

Additionally, we also examined the expression levels of IL-6, IL-1β, and TNF-α to further investigate specific inflammatory state in the SN and striatum (Figure 5). Similarly, acupuncture stimulation reduced the expression of pro-inflammatory cytokines in the SN (one-way ANOVA with *post hoc* Tukey’s tests: IL-6, *F*_4, 25_ = 20.6, *p* < 0.001; IL-1β, *F*_4, 25_ = 22.2, *p* < 0.001; TNF-α, *F*_4, 25_ = 26.7, *p* < 0.001; Figure 5A) and striatum (one-way ANOVA with *post hoc* Tukey’s tests: IL-6, F4, 25 = 54.1, *p* < 0.001; IL-1β, *F*_4, 25_ = 38.2, *p* < 0.001; TNF-α, *F*_4, 25_ = 48.6, *p* < 0.001; Figure 5B), but pretreatment with cromolyn or lidocaine blocked these effects. These findings suggest that the effect of acupuncture in ameliorating glial reactivity is mediated by both MC activation and nerve conduction.

### 3.5 Acupuncture stimulation at the GB34 changes local collagen fiber morphology

**Figure 6:**
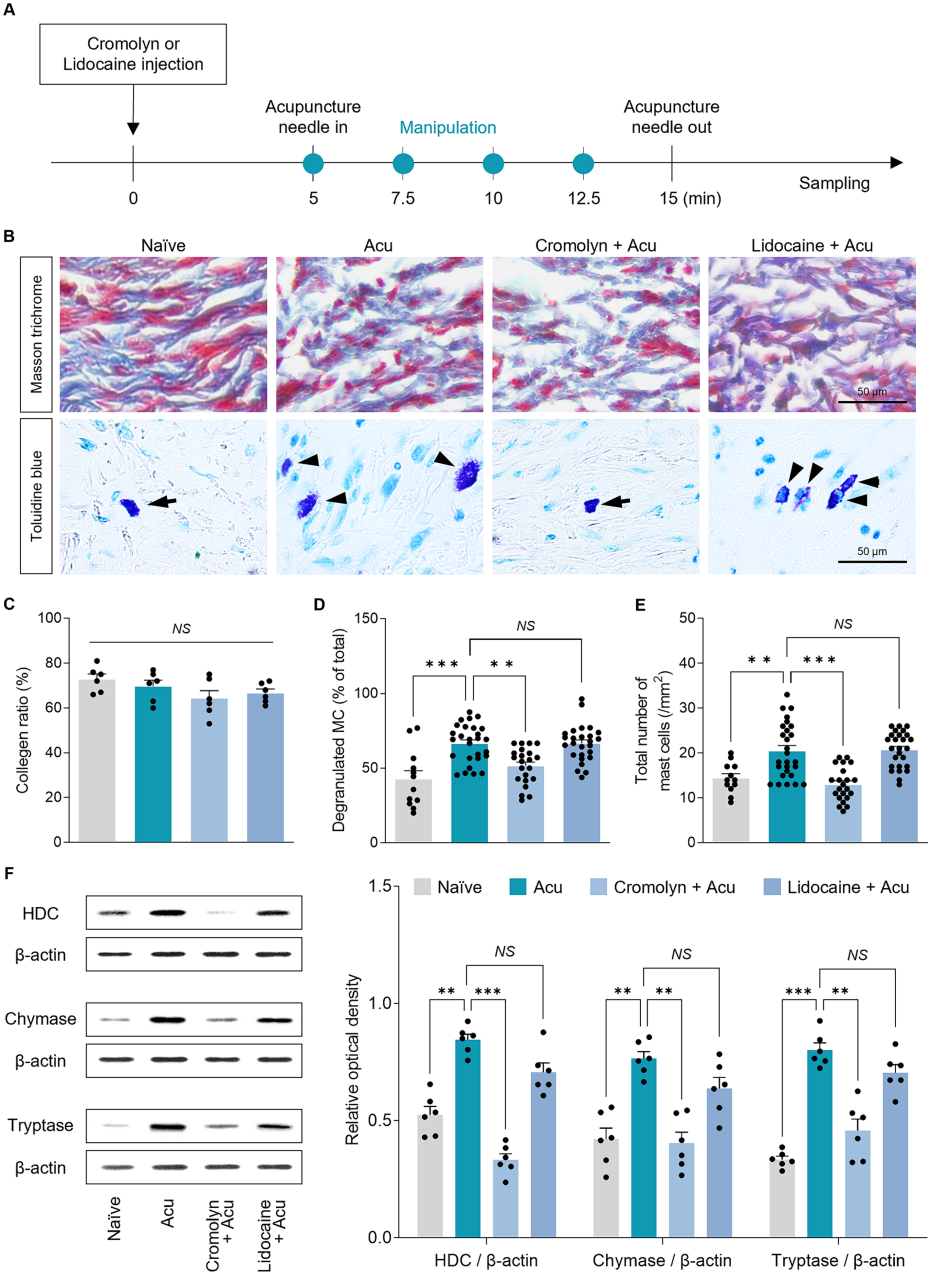
Peripheral MCs are upstream than nerves in the effects of acupuncture starting from acupoints. **(A)** Experimental timeline for identifying the causal relationship between MCs and nerves in acupoints. **(B)** Representative images of masson trichrome and toluidine blue staining in acupoints. **(C)** Collagen morphology and collagen ratio after acupuncture stimulation (*n* = 6 per group). **(D,E)** Quantification of number and degranulated peripheral MCs (*N* = 6 mice and *n* = 12–27 cells per group). **(F)** The expression levels of markers of MC degranulation by acupuncture stimulation in acupoints (*n* = 6 per group). ^***^p < 0.001, ^**^p < 0.01, ^*^p < 0.05. All data are presented as mean ± SEM.

To investigate the detailed mechanism at the acupoints, based on previous studies showing that acupuncture stimulation modifies collagen morphology at acupoints, we hypothesized that local collagen changes after acupuncture stimulation also occur in GB34 (Figure 6). Therefore, Masson’s trichrome staining was used to assess the morphology of collagen fibers or bundles in acupoints (Figure 6A). The parallel pattern of collagen bundles became irregular after acupuncture stimulation, and the pattern was similar even after pretreatment with cromolyn or lidocaine (Figure 6B, Top). However, there was no significant difference between each group in the amount of collagen by acupuncture stimulation (one-way ANOVA with *post hoc* Tukey’s tests: *F*_3, 20_ = 1.945, *p* = 0.155; Figure 6C). Therefore, it suggests that acupuncture stimulation alters local collagen morphology through mechanical stimulation, irrespective of peripheral MCs or nerves.

### 3.6 Acupuncture stimulation at the GB34 increases peripheral MC numbers and degranulation

MCs are activated when the collagen fibers are mechanically deformed. Thus, we investigated whether local MCs were activated in the GB34 acupoint where collagen was modified by acupuncture stimulation (Figure 6B, Bottom). We found that in the GB34 acupoint, MC numbers and degranulation were also increased by acupuncture stimulation (one-way ANOVA with *post hoc* Tukey’s tests: total number of MCs, *F*_3, 83_ = 16.53, *p* < 0.001; degranulated MC, *F*_3, 83_ = 13.30, *p* < 0.001; Figures 6D,E).

MCs are characterized by electrodense granules filled with potent mediators including HDC, chymase, and tryptase, which are released during degranulation. Therefore, we investigated whether MC degranulation increased mediator release during acupuncture stimulation. The release of mediators showed an increase due to an increase in MC degranulation during acupuncture stimulation at GB34 (one-way ANOVA with *post hoc* Tukey’s tests: HDC, *F*_3, 20_ = 49.27, *p* < 0.001; Chymase, *F*_3, 20_ = 16.51, *p* < 0.001; Tryptase, *F*_3, 20_ = 39.61, *p* < 0.001; Figure 6F). These results indicate that changes in MCs by acupuncture stimulation also occur at GB34, which supports the hypothesis that the number of MCs and the ratio of degranulation are increased by the mechanical stimulation of acupuncture at the acupoint.

### 3.7 Acupuncture signals precedes by MC activation rather than peripheral nerves

Finally, to study the causal relationship between peripheral MCs and nerve endings on acupuncture signals caused by needle stimulation, the number and degranulation ratio of MCs at GB34 acupoints were examined after pretreatment with cromolyn or lidocaine. The increase in the number of MCs and the degranulation ratio induced by acupuncture stimulation was blocked by cromolyn pretreatment, but not by lidocaine (one-way ANOVA with *post hoc* Tukey’s tests: total number of MCs, *F*_3, 83_ = 16.53, *p* < 0.001; degranulated MC, *F*_3, 83_ = 13.30, *p* < 0.001; Figures 6D,E). In addition, the increase in the release of MC mediators by acupuncture stimulation was blocked by pretreatment with cromolyn but not with lidocaine (one-way ANOVA with *post hoc* Tukey’s tests: HDC, *F*_3, 20_ = 49.27, *p* < 0.001; Chymase, *F*_3, 20_ = 16.51, *p* < 0.001; Tryptase, *F*_3, 20_ = 39.61, *p* < 0.001; Figure 6F). Together, these findings indicate that the effect of acupuncture initiated by needle stimulation on local acupoints is preceded by changes in MCs at the acupoints rather than in the nerves.

## 4 Discussion

**Figure 7:**
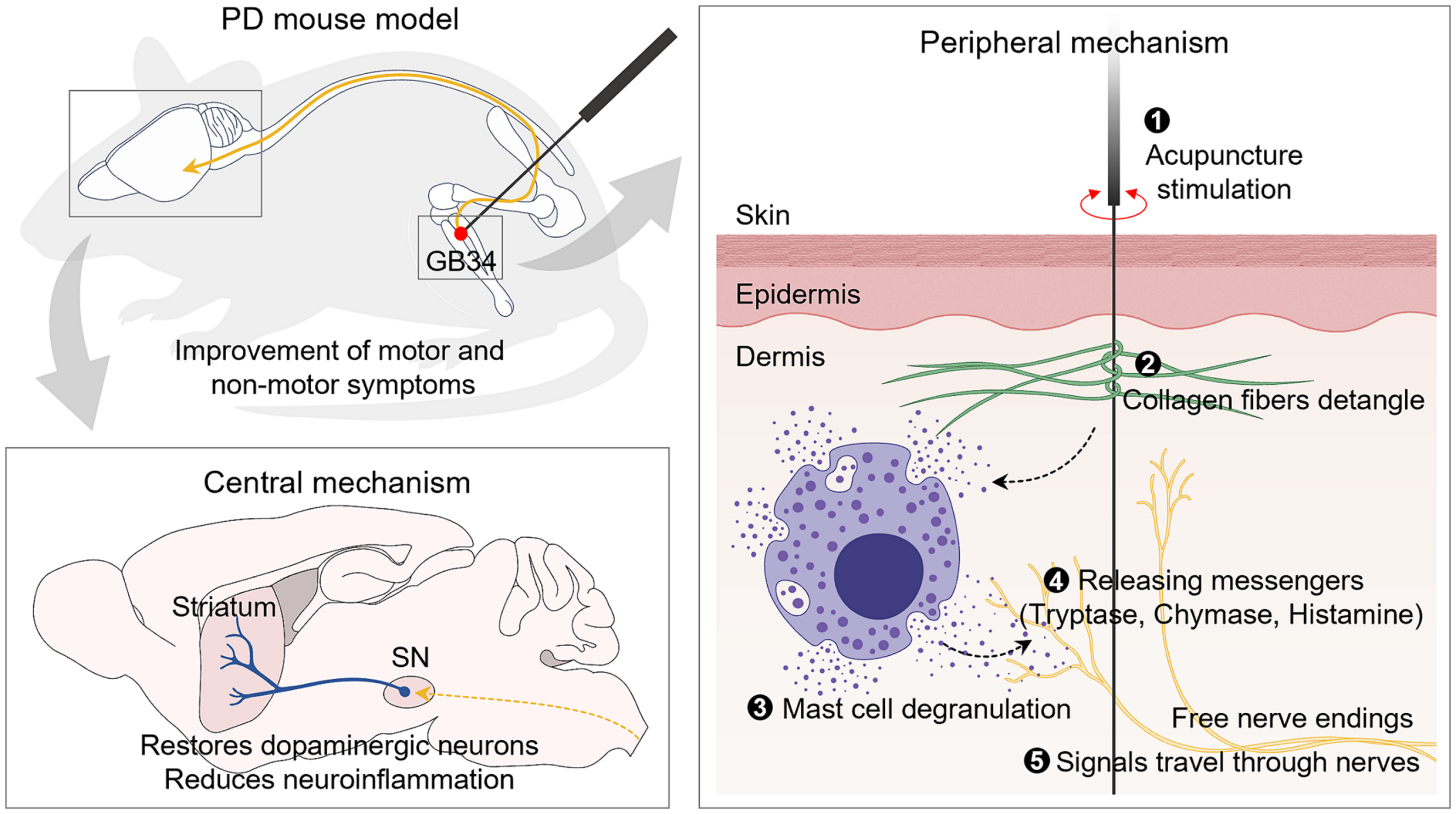
Schematic illustration of acupuncture signaling in an animal model of PD. Mechanical acupuncture stimulation on the GB34 acupoint modifies the morphology of collagen fibers and increases the number and degranulation ratio of MCs. As a result, the release of granules from MCs increases, and the signals are transmitted to nerves to exert dopamine neuroprotective effects in the SN and striatum. It also improves motor and non-motor deficits, which are typical symptoms of PD.

Despite the proven effectiveness of acupuncture in PD, research on the mechanisms in the periphery that generate acupuncture signals remains limited. Thus, our study aimed to elucidate the role of peripheral MCs in the effects of acupuncture on both motor and non-motor symptoms. Our findings revealed that acupuncture at GB34 increased the number and degranulation ratio of MCs at the acupoint in a PD mouse model. Importantly, we found that this increased degranulation ratio of MCs is crucial for initiating acupuncture signals, thereby contributing to the improvement of PD. This study suggests that MCs at acupoints may mediate the effects of acupuncture in a PD model, providing insights into the peripheral mechanism of acupuncture treatment for PD (Figure 7).

Notably, the population of MCs is denser at several acupoints (Mingfu et al., 2013), and acupuncture stimulation increases degranulation ratio of MCs (Zhang et al., 2008; Yu et al., 2009; Huang et al., 2012). Degranulation is the most characteristic change in MCs, which induces the exocytosis of pre-synthesized molecules from granules (Metcalfe et al., 1997). These released neuroactive molecules transmit acupuncture signals to various pathways, contributing to the overall acupuncture responses by interacting with the nervous, circulatory, and immune systems, resulting in acupuncture responses (Payne and Kam, 2004; Zhao et al., 2005; Parsons and Ganellin, 2006; de Souza Junior et al., 2015; Ding et al., 2018). Our study demonstrated that acupuncture at GB34 increased MC number and degranulation ratio at these acupoints. We also observed increased expression of MC degranulation markers such as HDC, chymase, and tryptase in response to acupuncture. Importantly, pretreatment with cromolyn, an MC stabilizer, suppressed the peripheral changes induced by acupuncture. Previous studies have suggested that acupuncture-induced MC degranulation is associated with the analgesic effects of acupuncture in models of inflammatory pain and adjuvant arthritis (Yu et al., 2008; Wu et al., 2015; Dimitrov et al., 2017). Nevertheless, studies addressing the role of peripheral MCs in brain-related diseases, including PD, have not been conducted.

Therefore, we explored whether peripheral MC activation via acupuncture plays a role in the effects of acupuncture in a mouse model of PD. First, we observed significant behavioral recovery and TH loss improvement in the SN and striatum following acupuncture treatment in the MPTP mouse model. However, pre-treatment with cromolyn around the acupoint before acupuncture inhibited the improvement of motor symptoms induced by acupuncture. Moreover, cromolyn pretreatment reduced the neuroprotective effects on dopaminergic neurons in the SN and striatum. These findings suggest that peripheral MCs play a crucial role in mediating the therapeutic effects of acupuncture on motor symptoms and dopaminergic neuroprotection in the SN and striatum.

It is important to note that non-motor symptoms such as anxiety, cognitive deficits, gastrointestinal dysfunction, sleep disorders, and motor symptoms are common features of clinical PD (Pfeiffer, 2016). Building on our previous study showing the positive effects of acupuncture on both motor deficits and anxiety-like behavior in PD mice (Jang et al., 2020), we investigated the role of peripheral MCs in the effects of acupuncture on anxiety-related behaviors. In the present study, acupuncture effectively ameliorated MPTP-induced anxiety. Notably, pretreatment of acupoints with cromolyn significantly reduced the anxiolytic effects of acupuncture, indicating the involvement of peripheral MCs in mediating the effects of acupuncture on non-motor symptoms. These findings suggest that the therapeutic effect of acupuncture on both motor and non-motor symptoms may be attributed to MC activation at the acupoint, resulting from the insertion and stimulation of acupuncture needles.

Glial cells are recognized as significant contributors to the neuroimmune response associated with various neurodegenerative pathologies and neurological symptoms (De Miranda et al., 2015; Lee et al., 2021). They show prominent activation in the SN and striatum following MPTP injection, suggesting that targeting and inhibiting glial activation could be a promising therapeutic strategy for alleviating PD symptoms. Therefore, our next objective was to investigate whether peripheral MCs modulate neuroinflammation and inflammatory cytokines in an MPTP model to understand their role in the therapeutic effects of acupuncture. Our findings revealed that acupuncture treatment effectively reduced MPTP-induced activation of astrocytes and microglia, as evidenced by decreased expression levels of GFAP and Iba-1 in the SN and ST, respectively. Additionally, acupuncture alleviated protein expression levels of proinflammatory cytokines such as IL-6, IL-1β, and TNF-α, indicating its potential anti-inflammatory effects in the model of PD. Interestingly, these beneficial changes were markedly blocked by pretreatment with cromolyn before acupuncture stimulation, suggesting a crucial role for peripheral MCs in mediating the effects of acupuncture on the regulation of neuroinflammation in the PD model.

To gain insight into how acupuncture activates MCs at acupoints, we examined histological observations of the tissue at the acupoint. These results confirmed that acupuncture induced the deformation of collagen fibers at acupoints, which subsequently led to MC degranulation. Previous studies have also reported that twisting or rotating acupuncture needle generates a winding effect on surrounding connective tissue, resulting in the deformation of collagen fibers (Langevin et al., 2001; Park et al., 2014). This may result in the degranulation of MCs. The significance of MC degranulation in acupuncture has been emphasized in earlier studies, and studies have shown that treating acupoints with collagenase reduces MC degranulation and diminishes the analgesic effects of acupuncture (Yu et al., 2008, 2009). In our study, although acupuncture manipulation altered the morphology of collagen fibers, pretreatment with cromolyn did not prevent this morphological alteration and inhibited the degranulation of MCs. Thus, we hypothesized that the mechanical signals induced by acupuncture may be responsible for the alteration of collagen fibers, ultimately leading to MC activation. However, further studies are needed to establish a definitive causal relationship between collagen fibers and MC degranulation at acupoints, and to clarify how acupuncture manipulation influences MC activation and its therapeutic effects.

To understand how altered MCs transmit signals to the brain, we explored the relationship between MCs and nerves. Acupuncture stimulation activates various afferent nerve fibers, converting them into nerve signals that are transmitted from afferent neurons to the central nervous system through the spinal cord (Zhao, 2008). In our study, peripheral nerve block with lidocaine suppressed the effect of acupuncture treatment in improving motor and non-motor deficits, as well as its anti-inflammatory actions in the SN and ST, indicating the involvement of afferent nerves around the acupoints in transmitting acupuncture signals. To gain a deeper understanding of the MC-nerve relationship, we further explored whether nerve blockade could suppress MC degranulation at acupoints. Our findings indicate that peripheral nerve blockade does not affect acupuncture-induced MC degranulation, supporting the notion that MCs may be located upstream of nerves at acupoints. These findings are consistent with previous research conducted in pain studies, where acupuncture at ST36 induced depolarization of sciatic nerves and dorsal root ganglia, which was inhibited by cromolyn administration (Sa et al., 2013); Although lidocaine administration abolished the analgesic effects of acupuncture, no discernible alteration in MC patterns induced by acupuncture manipulation was observed (Wang et al., 2022). Taken together, our study suggests that during acupuncture, mechanical stimulation activates MCs, and the mediators secreted by MCs enhance neural conduction, facilitating the transmission of acupuncture signals to the brain. However, further research is required to fully understand the intricate interplay between MCs and nerves in relation to the therapeutic effects of acupuncture.

This study has some limitations. While it sheds light on the roles of MCs at acupoints, further research is needed to explore the broader systemic effects and interactions between MCs and nerves under various physiological and pathological conditions. Although we observed changes in MC numbers and degranulation in response to acupuncture stimulation and nerve blockade, the specific signaling pathways and molecular mechanisms remain unclear. Thus, additional studies are needed to elucidate the intricate cellular and molecular interactions between nerves and MCs and the mechanisms of effects of acupuncture in PD. Despite these limitations, our study lays the groundwork for future research to advance acupuncture as a treatment for neurological conditions such as PD.

In conclusion, our study demonstrates the pivotal role of peripheral MCs and their activation at the GB34 acupoint in mediating the therapeutic effects of acupuncture in a mouse model of PD. Acupuncture stimulation led to an increase in the number and degranulation ratio of MCs as well as the expression levels of markers associated with MC activation, while cromolyn administration attenuated these effects and hindered the therapeutic benefits of acupuncture. These findings indicate the significance of peripheral MCs in ameliorating both motor and non-motor symptoms in patients with PD. Moreover, our study proposes a potential mechanism through which mechanical stimulation during acupuncture activates MCs and enhances neural conduction, facilitating the transmission of acupuncture signals to the brain. Overall, our research enhances understanding of the intricate interactions among acupuncture, MCs, and neural pathways, supporting the potential of acupuncture as a promising therapeutic intervention for PD and other neurological conditions.

## Data availability statement

The original contributions presented in the study are included in the article/Supplementary material, further inquiries can be directed to the corresponding author.

## Ethics statement

The animal study and procedures were conducted in accordance with the National Institutes of Health guidelines and approved by the Institutional Animal Ethical Committee, Kyung Hee University (Seoul, Korea, Approval Number KHSASP-23-398).

## Author contributions

J-YO: Conceptualization, Investigation, Methodology, Software, Visualization, Writing – original draft, Writing – review & editing. S-JB: Conceptualization, Investigation, Methodology, Writing – original draft. J-YJ: Investigation, Methodology, Writing – review & editing. T-YH: Investigation, Methodology, Writing – review & editing. SJ: Investigation, Methodology, Writing – review & editing. J-YP: Project administration, Validation, Writing – review & editing. S-NK: Project administration, Validation, Writing – review & editing. YR: Funding acquisition, Validation, Writing – review & editing. M-HN: Project administration, Validation, Writing – review & editing. H-JP: Conceptualization, Funding acquisition, Supervision, Validation, Writing – review & editing, Writing – original draft.
