# Supplementary material for: Peripheral mast cells derive the effects of acupuncture in Parkinson’s disease
Source: Front Aging Neurosci. 2024 Jun 24;16:1376756. doi: 10.3389/fnagi.2024.1376756 (PMC11229453; doi:10.3389/fnagi.2024.1376756)
Supplement: Supplementary file 1 [file Data_Sheet_1.PDF]

Supplementary Material

Peripheral mast cells derive the effects of acupuncture in Parkinson's disease

Ju-Young Oh, Sun-Jeong Bae, Jeong-Yeon Ji, Tae-Yeon Hwang, Suhwan Ji, Ji-Yeun Park, Seung-Nam Kim, Yeonhee Ryu, Min-Ho Nam, Hi-Joon Park*

*** Correspondence:** Hi-Joon Park: acufind@khu.ac.kr

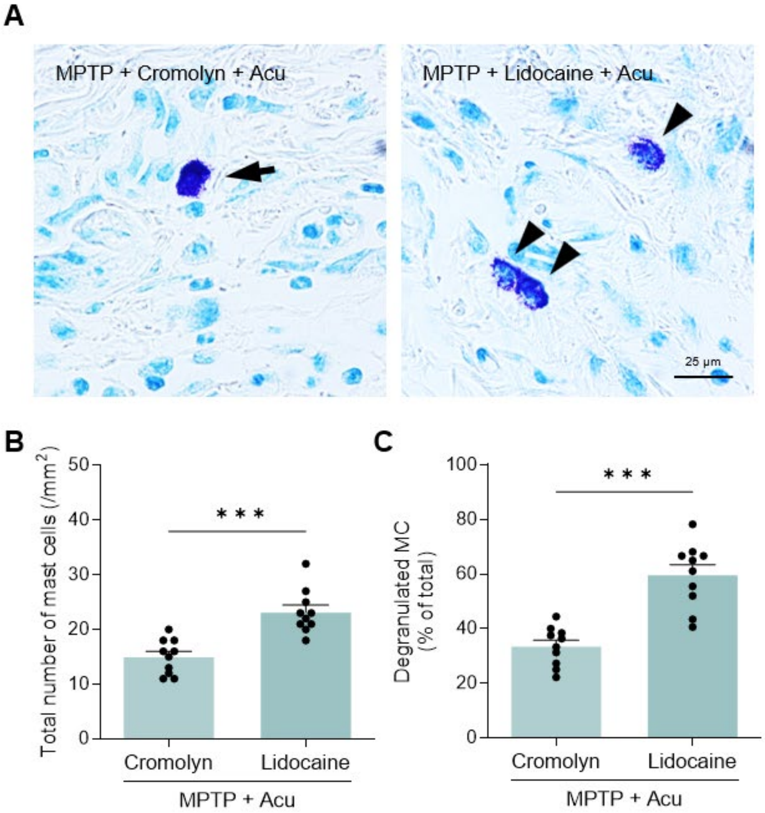

Supplementary Figure 1. The number and degranulation ratio of peripheral MCs associated with therapeutic effects of acupuncture in PD mice. (A) Representative images of toluidine blue staining for MCs. (B, C) Quantification of number and degranulated peripheral MCs (N = 5 mice and n = 10 cells per group). $^{***}p < 0.001$. All data are presented as mean ± SEM.
